# Influence of Emotional Intelligence, Motivation and Resilience on Academic Performance and the Adoption of Healthy Lifestyle Habits among Adolescents

**DOI:** 10.3390/ijerph16162810

**Published:** 2019-08-07

**Authors:** Rubén Trigueros, José M. Aguilar-Parra, Adolfo J. Cangas, Rosario Bermejo, Carmen Ferrandiz, Remedios López-Liria

**Affiliations:** 1 Department of Psychology, Hum-878 Research Team, Health Research Centre, University of Almería, 04120 Almería, Spain; 2 Department of Psychology, Hum-760 Research Team, Health Research Centre, University of Almería, 04120 Almería, Spain; 3 Department of Psychology, University of Murcia, 30100 Murcia, Spain; 4 Department of Nursing Science, Physiotherapy and Medicine, Hum-498 Research Team, Health Research Centre, University of Almería, 04120 Almería, Spain

**Keywords:** emotional intelligence, resilience, emotions, motivation, academic performance, physical activity

## Abstract

Included among the basic objectives of Physical Education (PE) classes is the consolidation of habits of a healthy lifestyle among adolescents. However, the main studies in this field have focused on cognitive aspects related to students during these classes, yet they ignore the role that emotions can play in the adoption of future habits. Objectives: To analyze how emotions (emotional intelligence and emotional state) can influence the resilience and motivation of adolescents, as well as academic performance and adoption of healthy lifestyle habits. Methodology: 615 secondary school students between the ages of 14 and 19 participated (*M* = 16.02; *SD* = 1.57) in the study. A structural equations model was developed using the main variables and by applying some of the principles of Self-Determination Theory. The results show that emotional intelligence is positively related to positive emotions and negatively related to negative emotions. Positive emotions positively predict both self-motivation towards physical education classes and resilience. Resilience positively predicts self-motivation. Finally, self-motivation acts as a predictor of both academic performance and regular participation in physical activity. Conclusions: This study successfully shows the importance of focusing on emotions in PE classes inasmuch as emotion increases the tendency to get good grades and maintain active lifestyle habits. In this sense, focusing on the emotions of students in PE could prove quite beneficial.

## 1. Introduction

According to the a World Health Organization report [1], 80% of the world’s population does not engage in any type of physical-sport activity (PA) regularly, despite the multiple psychological, physical and emotional benefits these activities provide. Such data are alarming considering sendentary lifestyles constitute a major risk factor for developing chronic illnesses, obesity, depression and anxiety [2]. In the case of Spain, the percentage of the population engaging in PA is around 41%, which is far removed from the approximate rate of 62% registered by northern European countries [3]. In addition, further WHO data [1] reveal that in Spain the prevalence of obesity increased from 3% to 12% among boys and from 2% to 8% among girls in the last ten years. Moreover, this source indicates that the highest rate of abandonment of PA occurs between the ages of 15 and 18, as this group’s interest changes to other leisure activities as they mature [4]. It is precisely for this reason that Physical Education (PE) classes represent the best “tool” for effectively consolidating active habits among young people that will endure throughout their lives. Indeed, according to the Organic Law for the Improvement of Educational Quality (LOMCE, in Spanish), one of PE’s main objectives is to teach healthy habits in relation to both food and responsible PA [5].

### 1.1. Emotional Intelligence

In recent years, Emotional Intelligence (EI) has become the focus of interest for numerous researchers. IE is understood as the ability to facilitate the recognition and regulation of emotions and the generation of adaptive behaviors [6]. The main theories on EI are based on the trait model [7] and the ability model [8], which share several common elements such as the fact that emotions are considered predictors of positive adaptive behaviors [6].Trait theory considers EI to be a construct that is linked to a set of stable traits related to personality, socio-emotional skills, motivational aspects and various cognitive abilities that are essential for facing demands and pressures [9]. The ability model views EI as another type of intelligence based on the adaptive use of emotions and their application to our thinking, allowing the individual to adapt to their surroundings and resolve problems.

According to BarOn [10], the emotional intelligence of an individual evolves in parallel with age, due to the influence of a set of social, psychological, affective and personal factors that influence a person’s capacity to adapt effectively to possible vicissitudes at the behest of the environment. For this reason, various studies in the field of education and developmental, EI has been positively linked to better psychological well-being of secondary students [11], emotional well-being [6], academic performance, social relationships [12], self-efficiency and empathy [13]. In contrast, EI has been negatively associated with stress [11], depression [14] and negative emotions [9].

### 1.2. Emotions

Emotions describe and explain the phenomena produced through a multidimensional process responsible for analyzing and interpreting specific situations, expression of emotion, preparation for action and, finally, the physiological and cognitive changes derived from the overall process [15]. Emotions have a social adaptation and personal adjustment function [16]. The emotional response of the individual is subordinated to three dimensions that organize all emotional states [17]: valence (the pleasant or unpleasant nature of an emotion that drives a person to avoid or approach the stimulus of origin), arousal (the strength of the emotional response, related to the intensity of the stimulus or internal motivation), and power (ranges from the extreme of dominance of a situation to submission).

According to various studies in the educational field, positive emotions have been significantly linked to academic ability and well-being [18], intrinsic motivation [19], participation [20] and memory [21]. Negative emotions have been positively linked to poor academic performance and dropout [22], levels of stress and depression [23]. As for the level of arousal, studies focused on anxiety have observed that this emotion plays a protector role [24]. Adequate levels of anxiety have been significantly linked to the academic performance and motivation of students [24], while both high and low levels of anxiety displayed negative relationships.

### 1.3. Resilience

PE classes are characterized by continuous exposure to a series of adverse and potentially stressful circumstances that each student must face to a certain degree at some point (e.g., complicated motor skill actions, physical contact with classmates and injuries). It is precisely these adverse situations that tend to be characterized in terms of “acute” and “chronic” adversities that are seen as “major aggressions” to developmental processes [25]. However, over time, the individual will readjust and regain balance, thus raising his or her level of resilience or homeostasis [25]. In this way, resilience has been defined as the process, capacity, or outcome of successful adaptation despite challenging or threatening circumstances [26]. In an academic context, resilience is understood as the student’s ability to successfully overcome possible environmental adversities caused by early traits, conditions, and experiences [27]. To this end, it is essential that students maintain a high level of intrinsic motivation and performance despite the presence of stressful events and conditions that result in poor performance in school and ultimately in dropping out of school [26].

On the other hand, Fletcher & Sakar [28] conceptualized the notion of resilience, defining it as the possession and presence of factors of vulnerability and protection, both inside and outside the individual, that influence positive adaptation to risk. These protective aspects of resilience have been examined in numerous studies which attempt to identify the qualities of resilient individuals in the fields of healthcare [29], the military [30], the workplace [31], and sports [32]. However, there are few works in the field of education (e.g., Salavera, Usán & Jarie, [33]), and those that exist primarily focus on aspects that are external to students and not on what transpires during classes.

### 1.4. Self-Determination Theory

This theory attempts to explain the influence of personal and social surroundings on human behavior, highlighting three different types of motivation: amotivation (complete lack of interest in engaging in an activity), controlled motivation (lack of commitment and active participation with the absence of external stimulus) and self-motivation (when individuals persist and adhere to a series of behaviors without the need for external stimuli, linked to a sense of their own choice and action) [34].

Types of self-motivation include intrinsic motivation (participation in an activity for the inherent satisfaction and enjoyment), integrated regulation (participation is related to the “inner self”) and identified regulation (behavior is motivated by internal goals). Non-autonomous forms of motivation include introjected regulation (participation in activities to avoid feelings of guilt or shame) and external (linked to social recognition and/or to obtaining some reward). Demotivation refers to the lack of initiative to participate in an activity due to a lack of apparent, internal or external incentive.

Self-Determination Theory (SDT) has been applied in numerous contexts (e.g., the workplace, physical activity, education, counseling), and studies have observed the notable influence of self-motivation on the adoption of adaptive behaviors [35], psychological well-being [36], social development [37], and self-esteem [38].

### 1.5. Justification

Until now, scientific literature has mainly focused on the motivational processes of students and their influence on decision-making, without taking into consideration the influence that emotions and resilience may have [39]. Therefore, the present study seeks to analyze, within the context of PE, the influence of emotional intelligence on the emotional state of adolescents during classes. The subject of PE is selected as it could especially contribute towards the development of social and emotional intelligence because it is an ideal context for conveying feelings and emotions that humanize personal contact through physical activity [40]. Additionally, this work aims to determine how emotions influence the resilience of these students, given that during PE classes they may experience a variety of emotions depending on their preferences and needs, their relationships with classmates, their experiences with the exercises themselves, the influence of the teacher and other factors, both personal and social, which may generate an adaptive or disadaptive behavioral response. In this line, a study conducted by Greco, Morelato and Ison [41] with nursery school students using a preliminary evaluation revealed that the positive emotional response produced during a situation external to the individual could generate an adaptive behavioral response in the future when the given surroundings so demand.

Finally, the present study also seeks to analyze how resilience could influence the motivation of students towards PE classes, and how the latter may influence academic performance and the intention to be physically active in the future. However, resilience is not a factor that has been studied in depth in the field of PE classes [42], despite being considered an internal attribute that contributes to individuals’ adaptive capacities against any possible difficulties they may encounter, resulting in positive adaptive behaviors (e.g., in sport [28]). According to SDT, these inner powers are closely linked to the internal motivation within subjects responsible for awakening energizing, directing and maintaining behavior and effort towards adaptive behaviors, which could also result in improved academic performance and the adoption of healthy habits [42]. In this sense, this result agrees with the postulates established by SDT, in which a study by Hagger and Chatzisarantis [43] established that intrinsic motivation towards PE classes positively predicted the practice of physical activity. Both contexts are closely related since, among the goals of PE classes, are the acquisition of active habits outside of the school setting [44,45].

### 1.6. Objective and Hypothesis

**Figure 1:**
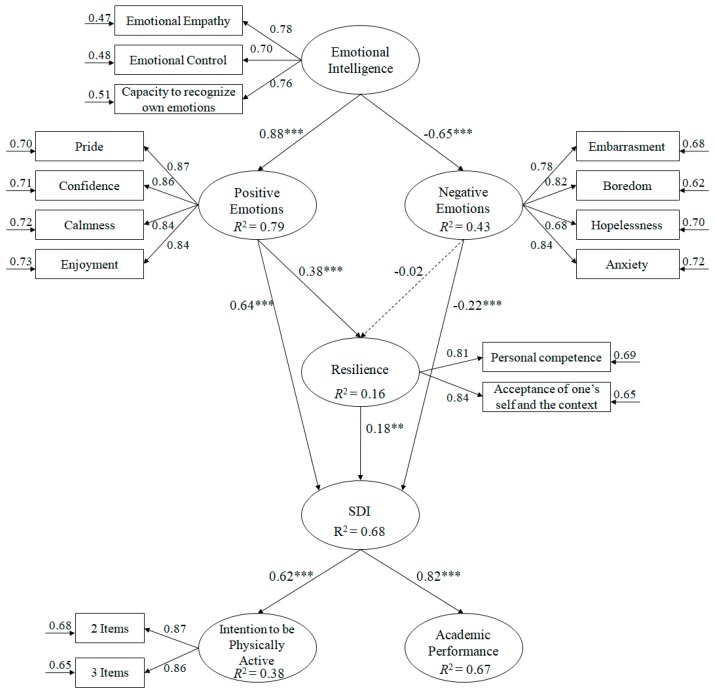
Hypothesized model, where all variables are related to one another. All parameters are standardized and statistically significant. Note: *** *p* < 0.001; ** *p* < 0.01.

The objective of the study was to analyze in the context of Physical Education (see Figure 1) the influence of emotional intelligence on emotional state, self-motivation and resilience among secondary school students, as well as on their academic performance and intention to adopt active habits of physical activity outside of school. The hypotheses formulated were: (1) Emotional intelligence will positively predict positive emotions and negatively predict negative emotions; (2) positive emotions will positively predict resilience and motivation; (3) negative emotions will negatively predict resilience and motivation; (4) resilience will positively predict motivation; and (5) motivation will positively predict both the intention to be physically active and academic performance.

## 2. Method

### 2.1. Participants

A total of 615 secondary school students participated in the study (318 boys and 297 girls). These participants were between the ages of 14 and 19 (M = 16.02; DT = 1.57) and belonged to various schools in a province of Spain. The students attended two PE classes, each with a duration of one hour. The classes were conducted with respect to the equality of both students’ rights and duties.

### 2.2. Instruments

Emotional Intelligence: The instrument utilized was the Spanish version of the Emotional Intelligence Scale in PE by Cecchini et al. [6], taken from the version by Arruza et al. [46]. This questionnaire features 22 items distributed among three factors which measure the capacity to recognize one’s own emotions (e.g., It is easier for me to recognize my emotions during activities), regulation and emotional control (e.g., when I face a game and/or competition, I control my emotions) and emotional empathy (e.g., I easily understand how my teammates or rivals feel in games and competitions). The responses to the different items were based on a Likert scale from 1 (totally disagree) to 7 (totally agree).

Emotions: The tool used was the Questionnaire on Emotional State in PE (QESP) developed by Trigueros, Aguilar-Parra, Cangas, Gallego and López-Liria [47]. The questionnaire features 34 items distributed among eight factors, four of which relate to positive emotions (e.g., I usually find the exercises to be interesting) and the other four to negative emotions (e.g., I feel frustrated and useless). The students responded based on a Likert scale from 1 (totally disagree) to 7 (totally agree).

The fit indices displayed by the scale were adequate [47]: *χ^2^/df* = 2.04, *p* = 0.001; Comparative Fit Index (CFI) = 0.94; Taylor Lewis Index (TLI) = 0.94; Root Mean Square Error of Approximation (RMSEA) = 0.048; Standardized Root Mean Square Residual (SRMR) = 0.048. The correlation between the factors ranged between −0.35 and 0.84, all of which were statistically significant. The correlation was positive between factors with the same valence, and negative between those with a different valence. The Cronbach’s alpha values for the factors were greater than 0.70.

Resilience: This aspect was measured using the Scale on Resilience in PE classes by Trigueros, Navarro, Aguilar-Parra, Ferrándiz, and Bermejo [48]. This questionnaire displays the heading “Considering your experiences during PE classes, indicate to what extent you agree or disagree with the following statements”. The scale features 25 items distributed between two factors that measure personal competence (e.g., when I want to overcome a challenge, I continue to do so) and acceptance of one’s self and the context (e.g., rarely do I think I will fail to achieve my goals). The students responded based on a Likert scale from 1 (totally disagree) to 7 (totally agree).

Motivation: The instrument used was the Spanish version of the Perceived Locus of Causality Revised (PLOC-R) by Vlachopoulos et al. [49], validated and adapted to a PE context in Spain by Trigueros et al. [5]. The scale features 23 items grouped among six factors that measure intrinsic motivation (e.g., because PE is fun), integrated regulation (e.g., because it is according to my way of life), identified regulation (e.g., because it is important to me to try in PE), introjected regulation (e.g., because it would bother me if I did not), external regulation (e.g., because that is the rule) and amotivation (e.g., but I really do not know why). The students responded utilizing a Likert scale ranging from 1 (not true at all) to 7 (totally true).

The self-determination index (SDI; [50]) was utilized. The SDI was calculated based on the following formula: 3 × intrinsic motivation, 2 × integrated regulation, 1 × identified regulation, −1 × introjected regulation, −2 × external regulation and −3 × demotivation. This index has been proven to be valid and reliable in several works, in which it was utilized to obtain a value that made it possible to quantify the level of self-determination.

Intention to be physically active: The tool used was the Spanish version of the Intention to Be Physically Active by Hein, Müür, and Koka [51], validated and adapted to the PE context in Spain by Moreno, Moreno, and Cervelló [52]. This scale is comprised of five items (e.g., Outside PE lessons I like to do sport) which measure intention. Students responded to a Likert scale whose responses ranged from 1 (totally disagree) to 7 (totally agree).

Academic performance. This aspect was measured by taking the grades obtained by students during the first term of PE class. The grades were distributed in the following way: 0 (fail) 1 (pass), 2 (good), 3 (great), and 4 (outstanding).

### 2.3. Procedure

Initially, as the students were under age, a written authorization was requested from both the school and the parents of the participants. Beforehand, teachers were informed that the questionnaires would be administered before the beginning of PE classes. Subsequently, students were told that they would be participating in a research investigation on motivation and emotions towards PE classes. The questionnaires were answered anonymously. This study was carried out in accordance with the recommendations of the American Psychological Association. The entire experiment was conducted in accordance with the Declaration of Helsinki. Ethics approval was obtained from the Research Ethics Committee of the University of Almeria, Spain (Ref. UALBIO 2019/014).

### 2.4. Data Analysis

The present study carried out descriptive statistical analyses, bivariate correlations and reliability analyses using the statistics program SPSS v25 (IBM, Armonk, NY, USA). In addition, a structural equations model was analyzed (SEM) using statistics the program AMOS v20 (IBM, Armonk, NY, USA).

In order to analyze the hypothesized model (Figure 1) the method utilized was the maximum likelihood estimation, in conjunction with a bootstrapping procedure. The estimators were not affected and were therefore considered robust. Aiming to accept or reject the tested model, a set of fit indices were taken into consideration [53]: χ^2^/gl, CFI (Comparative Fit Index), IFI (Incremental Fit Index), RMSEA (Root Mean Square Error of Approximation) plus its Confidence Interval (IC) at 90%, and SRMR (Standardized Root Mean Square Residual). Given that the χ^2^/gl values were below 3, the values for the incremental indices (CFI, IFI) were close to or greater than 0.95 and the RMSEA and the SRMR values were lower than or close to 0.06 and 0.08, it was considered that the data displayed good fit to the model [48]. However, Marsh, Hau, and Wen [54] stated that these cut-off values should be interpreted with caution as they may prove too restrictive and difficult to obtain when complex models are tested.

## 3. Results

### 3.1. Preliminary Analysis

Table 1 displays the mean and standard deviation, bivariate correlations, and reliability analysis using Cronbach’s α of emotional intelligence, positive and negative emotions, resilience, self-determination index, intention to be physically active and academic performance.

The results of the correlation analyses, the results revealed a positive association between EI and positive emotions, self-determined motivation, resilience, academic performance and intention to be physically active. Positive emotions demonstrated a negative association with regard to negative emotions, but the association with the rest of the variables was positive. Negative emotions showed a negative association with all the variables in the study. Resilience revealed a positive correlation with self-determined motivation, academic performance, and intention to be physically active. Finally, self-determined motivation reflected a positive association with academic performance and the intention to be physically active, in which the correlation between the latter two was positive.

### 3.2. Structural Equation Model

Prior to testing the hypothesized model using an SEM and analyzing the relationships existing between the variables belonging to the model, the number of latent variables was reduced, whereby each one had at least two indicators [55]. The hypothesized model for the predictive relationships (Figure 1) showed that the fit indices were adequate: χ^2^ (80, N = 615) = 259.44, χ^2^/gl = 3.24, *p* < 0.001, IFI = 0.95, CFI = 0.95, RMSEA = 0.062. (IC 90% = 0.057−0.069), SRMR = 0.057. The results fitted the established parameters, meaning that the proposed model was accepted as suitable. Similarly, the contribution of each one of the factors to the prediction of the other variables was examined by means of standard regression weights.

## 4. Discussion

This study analyzed, in the context of Physical Education, the influence of emotions (emotional intelligence and emotional state) on motivation and the resilience of secondary school students, as well as on their academic performance and intention to adopt physical activity habits outside of school.

The findings reveal that emotional intelligence is positively related to positive emotions and negatively to negative emotions, precisely as cited in previous university studies in the field of academia. Such studies confirmed the negative effect of emotional intelligence in relation to emotions like anxiety [14,56] and its positive effect on emotions like happiness [33]. However, there is hardly any evidence from investigations that have analyzed the influence of emotional intelligence or the emotional state experienced by students themselves during PE classes, despite the importance of this subject in terms of conveying and experiencing multiple emotions [57]. In this sense, the results of the present study underscore the key role of emotional intelligence in terms of regulating and repairing emotions and being emotionally efficient, which leads to greater emotional satisfaction and produces a stronger sense of emotional well-being and a healthier mentality. In turn, these benefits afford individuals the chance to be better adjusted, both psychologically and in terms of character [22].

The results also demonstrated that positive emotions positively predicted self-motivation towards physical education classes and resilience. However, negative emotions negatively predicted self-motivation and resilience. The latter was not statistically significant, except with the Pearson correlation analysis. It is difficult to compare these results with those of other studies as research on emotions in education is still in a state of relative fragmentation [9]. For example, a study carried out by Mega et al. [19] demonstrated that the influence of positive emotions among students improves their confidence in their own abilities, increases their intrinsic motivation towards learning, and their intention to perform certain actions. Furthermore, positive emotions had a higher explanation rate than negative emotions in relation to motivation. These results highlighted and demonstrated the relevance of positive emotions in relation to student involvement and indeed towards motivation, which underscores the premise established by Pekrun, Lichtenfeld, Marsh, Murayama, and Goetz [19] that positive emotions should be worked on and strengthened in academic surroundings for the purpose of favoring students’ motivation and learning. Additionally, a study conducted by Tugade et al. [23] revealed how a positive emotional state positively influenced an individual’s resilience by acting as a protective factor, as a positive emotional state tends to improve the coping process when dealing with stressful moments by diminishing the influence of external factors that attempt to distract the student [28].

The results of the present study show that resilience positively predicts self-motivation. However, studies on resilience in the field of PE classes are rather scarce and almost nonexistent. Despite this situation, in the university context, Magnano, Craparo, and Paolillo [58] analyzed the influence of resilience on self-motivation and found that students who were psychologically strong (resilient) utilized internal coping strategies which led them to acutely perceive, access, and regulate their behaviors in order to achieve their objectives (self-motivation).

Finally, the present study shows how self-motivation acts as a predictor of both academic performance and regular participation in physical activity. These results are in accordance with various studies [59,60] in which high levels of self-motivation possessed by certain students have a positive influence on the adoption of healthy lifestyle habits, such as regular participation in physical activity outside of school [61]. Similarly, several studies have demonstrated that those students who possess high levels of self-motivation display greater involvement in classes and make better decisions, resulting in high academic performance [62].

The present research has confirmed that academic performance has a higher percentage of explained variance (*R*^2^) than physical activity habits, which indicates that PE students display a greater tendency towards obtaining good grades than interest in developing active lifestyle habits. In this sense, this result could be explained by the fact that P.E. students show more interest in the factors present that affect them now (grades in a class) rather than in the foreseeable future (intentions to lead an active lifestyle later) [63]. Consequently, secondary school students concentrate their efforts and attention on obtaining good grades, to the detriment of unrelated attitudes, content and values that are beneficial to active lifestyle habits.

Despite the benefits described in the model, there are several limitations to the study, which must be highlighted in order to generalize the results. Firstly, the present work relies primarily on self-report measures. In addition, it presents an assessment of students’ grades in a single course. Secondly, the fact that this is a relational study does not allow us to extrapolate cause-effect relationships cannot be extrapolated and the results obtained could be interpreted in a variety of different ways, depending on the perspective of the individual. Thirdly, the relationship established between negative emotions and resilience through the SEM was not significant, implying that future research should analyze this relationship more in depth using similar and/or different populations. However, the model seems to show good robustness. Future research should perform an ethnographic study with the goal of delving deeper into and understanding the psychological aspects of students. Furthermore, future lines of research could focus on how teachers, friends and family may influence the development of students’ emotional intelligence, motivation and resilience and to what extent said influence remains stable over time. In addition, it would also be useful to determine the influence of motivation on the resilience, given the variability of how adolescent are perceived as they grow up and progressively make their own decisions independently. Therefore, PE teachers should design educational programs that focus on the development of effective relationships, recognition of negative and positive emotions, and interpersonal communication, not only on developing physical sports skills, knowledge, techniques, and strategies [64].

## 5. Conclusions

These results suggest that emotional intelligence exerts a positive influence on positive emotions, and a negative influence on negative emotions. Furthermore, positive emotions exert a positive influence on resilience and self-motivation. In contrast, negative emotions exert a negative influence on self-motivation and resilience, although the latter is not statistically significant. Finally, resilience exerts a positive influence on self-motivation, and motivation has a positive influence on academic performance and the adoption of physical activity habits. This study successfully shows the importance of focusing on emotions from PE classes. In this sense, giving a greater presence to emotions in the classroom is one of the important future tasks within the current educational system, as it has traditionally prioritized cognitive development over affective and emotional development.

This prioritization has led to enormous costs of emotional illiteracy centred on the recognition and management of students’ emotions. This emotional illiteracy has had a negative impact on motivation [57], coping, academic performance, and the adoption of positive adaptive behaviors [37,56,57].

## Figures and Tables

**Table 1:** Descriptive statistics and correlations between all the variables.

Factors	*M*	*SD*	AVE	α	1	2	3	4	5	6	7
1.Emotional intelligence	3.10	0.84	0.67	0.76	-	0.73 ***	−0.66 ***	0.28 ***	0.70 ***	0.24 ***	0.60 ***
2. Positive emotions	4.94	1.51	0.80	0.92		-	−0.79 ***	0.35 ***	0.79 ***	0.27 ***	0.72 ***
3. Negative emotions	2.55	1.49	0.69	0.93			-	−0.29 *	−0.74 ***	−0.13 ***	−0.55 ***
4. Resilience	3.27	0.72	0.84	0.82				-	0.36 ***	0.18 ***	0.33 ***
5. SDI	11.56	16.02	-	-					-	0.28 ***	0.82 ***
6. IPA	3.11	0.66	0.72	0.95						-	0.35 ***
7.Academic performance	1.94	1.32	-	-							-

Note: AVE = Average Variance Extract; M = Mean; SD = Standard Deviations; SDI = Self-Determination Index; IPA = Intention to be Physically Active; *** *p* < 0.001; * *p* < 0.05; *N* = 615.

## References

[1] Organización Mundial de la Salud (2018). Actividad Física.

[2] Standal Ø.F., Aggerholm K. (2016). Habits, skills and embodied experiences: A contribution to philosophy of physical education. Sport Ethics Philos..

[3] Ministerio de Educación, Cultura y Deporte (MECD) (2015). Anuario de Estadísticas Deportivas. Encuesta Sobre los Hábitos Deportivos 2015.

[4] Ntoumanis N. (2012). A self-determination theory perspective on motivation in sport and physical education: Current trends and possible future research directions. Motiv. Sport Exerc..

[5] Trigueros R., Sicilia A., Alcaraz-Ibáñez M., Dumitru D.C. (2017). Spanish adaptation and validation of the Revised Perceived Locus of Causality Scale in physical education. Cuadernos de Psicología del Deporte..

[6] Cecchini J.A., Méndez-Giménez A., García-Romero C. (2018). Validación del Cuestionario de Inteligencia Emocional en Educación Física. Revista de Psicología del Deporte..

[7] Bar-On R. (2006). The Bar-On model of emotional-social intelligence (ESI). Psicothema..

[8] Mayer J.D., Salovey P., Caruso D.R. (2004). Emotional intelligence: Theory, findings and implications. Psychol. Inq..

[9] Pekrun R., Linnenbrink-Garcia L. (2012). Academic emotions and student engagement. Handbook of Research on Student Engagement.

[10] BarOn R. (2010). Emotional intelligence: An integral part of positive psychology. South Afr. J. Psychol..

[11] Por J., Barriball L., Fitzpatrick J., Roberts J. (2011). Emotional intelligence: Its relationship to stress, coping, well-being and professional performance in nursing students. Nurse Educ. Today..

[12] Song L.J., Huang G.H., Peng K.Z., Law K.S., Wong C.S., Chen Z. (2010). The differential effects of general mental ability and emotional intelligence on academic performance and social interactions. Intelligence..

[13] Kokkinos C.M., Kipritsi E. (2012). The relationship between bullying, victimization, trait emotional intelligence, self-efficacy and empathy among preadolescents. Soc. Psychol. Educ..

[14] Dewaele J.M., Petrides K.V., Furnham A. (2008). Effects of trait emotional intelligence and sociobiographical variables on communicative anxiety and foreign language anxiety among adult multilinguals: A review and empirical investigation. Lang. Learn..

[15] Nakamura J., Csikszentmihalyi M. (2014). The concept of flow. Flow and the Foundations of Positive Psychology.

[16] Schutz P.A., Pekrun R. (2007). Introduction to emotion in education. Emotion in Education.

[17] Faith M., Thayer J.F. (2001). A dynamical systems interpretation of a dimensional model of emotion. Scand. J. Psychol..

[18] Schonert-Reichl K.A., Lawlor M.S. (2010). The effects of a mindfulness-based education program on pre-and early adolescents’ well-being and social and emotional competence. Mindfulness..

[19] Pekrun R., Lichtenfeld S., Marsh H.W., Murayama K., Goetz T. (2017). Achievement emotions and academic performance: Longitudinal models of reciprocal effects. Child Dev..

[20] Mega C., Ronconi L., De Beni R. (2014). What makes a good student? How emotions, self-regulated learning, and motivation contribute to academic achievement. J. Educ. Psychol..

[21] Winter D., Elzinga B., Schmahl C. (2014). Emotions and memory in borderline personality disorder. Psychopathology..

[22] Nolen-Hoeksema S., Aldao A. (2011). Gender and age differences in emotion regulation strategies and their relationship to depressive symptoms. Personal. Individ. Differ..

[23] Tugade M.M., Fredrickson B.L., Feldman Barrett L. (2004). Psychological resilience and positive emotional granularity: Examining the benefits of positive emotions on coping and health. J. Personal..

[24] Yli-Piipari S., Watt A., Jaakkola T., Liukkonen J., Nurmi J.E. (2009). Relationships between physical education students’ motivational profiles, enjoyment, state anxiety and self-reported physical activity. J.Sports Sci. Med..

[25] Richardson G.E. (2002). The metatheory of resilience and resiliency. J. Clin. Psychol..

[26] Martin A.J., Marsh H.W. (2009). Academic resilience and academic buoyancy: Multidimensional and hierarchical conceptual framing of causes, correlates and cognate constructs. Oxf. Rev. Educ..

[27] Martin A.J. (2013). Academic buoyancy and academic resilience: Exploring everyday and classic resilience in the face of academic adversity. Sch. Psychol. Int..

[28] Fletcher D., Sarkar M. (2012). A grounded theory of psychological resilience in Olympic champions. Psychol. Sport Exerc..

[29] Heath M.A., Donald D.R., Theron L.C., Lyon R.C. (2014). AIDS in South Africa: Therapeutic interventions to strengthen resilience among orphans and vulnerable children. Sch. Psychol. Int..

[30] Bartone P.T. (2006). Resilience under military operational stress: Can leaders influence hardiness?. Mil. Psychol..

[31] MacEachen E., Polzer J., Clarke J. (2008). You are free to set your own hours: Governing worker productivity and health through flexibility and resilience. Soc. Sci. Med..

[32] Galli N., Vealey R.S. (2008). Bouncing back from adversity: Athletes experiences of resilience. Sport Psychol..

[33] Salavera C., Usán P., Jarie L. (2017). Emotional intelligence and social skills on self-efficacy in Secondary Education students. Are there gender differences?. J. Adolesc..

[34] Ryan R.M., Lynch M.F., Vansteenkiste M., Deci E.L. (2011). Motivation and autonomy in counseling, psychotherapy, and behavior change: A look at theory and practice 1ψ7. Couns. Psychol..

[35] Sun H., Chen A. (2010). A pedagogical understanding of the self-determination theory in physical education. Quest..

[36] Hancox J.E., Ntoumanis N., Thøgersen-Ntoumani C., Quested E. (2015). Self-Determination theory. Europe Active’s Essentials of Motivation and Behavior Change for Fitness, Health and Sport Professionals.

[37] Keatley D., Clarke D.D., Hagger M.S. (2012). Investigating the predictive validity of implicit and explicit measures of motivation on condom use, physical activity and healthy eating. Psychol. Health..

[38] Deci E.L., Ryan R.M., Ryan R.M. (2012). Motivation, personality and development with in embedded social contexts: An overview of self-determination theory. The Oxford Handbook of Human Motivation: Oxford.

[39] Bekerman Z., Zembylas M. (2018). Emotion, Emotional Intelligence and Motivation. Psychologized Language in Education.

[40] Del Estado J. (2013). Ley Orgánica 8/2013, de 9 de diciembre, para la Mejora de la Calidad Educativa (LOMCE).

[41] Greco C., Morelato G., Ison M. (2007). Emociones positivas: Una herramienta psicológica para promocionar el proceso de resiliencia infantil. Psicodebate..

[42] Trigueros R., Aguilar-Parra J.M., Cangas A.J., López-Liria R., Álvarez J.F. (2019). Influence of Physical Education Teachers on Motivation, Embarrassment and the Intention of Being Physically Active During Adolescence. Int. J. Environ. Res. Public Health..

[43] Hagger M., Chatzisarantis N. (2016). The trans-contextual model of autonomous motivation in sport: Conceptual and empirical issues and meta-analysis. Rev. Educ. Res..

[44] Silva D.A.S., Chaput J.P., Katzmarzyk P.T., Fogelholm M., Hu G., Maher C., Tudor-Locke C. (2018). Physical education classes, physical activity, and sedentary behavior in children. Med. Sci. Sports Exerc..

[45] Lander N., Eather N., Morgan P.J., Salmon J., Barnett L.M. (2017). Characteristics of teacher training in school-based physical education interventions to improve fundamental movement skills andor physical activity: A systematic review. Sports Med..

[46] Arruza J.A., González O., Palacios M., Arribas S., Telletxea S. (2013). Un modelo de medida de la inteligencia emocional percibida en contextos deportivo/competitivos. Revista de Psicología del Deporte..

[47] Trigueros R., Aguilar-Parra J.M., Álvarez J.A., Cangas A.J., Gallego J., López-Liria R. (2019). Validation of the Scale of Emotional States in the Physical Education Context. Front. Psychol..

[48] Trigueros R., Navarro N., Aguilar-Parra J.M., Ferrándiz C., Bermejo C. (2019). Adaptación y Validación de la Escala de Resiliencia en el Contexto de la Actividad Física al Contexto de la Educación Física. Apunts.

[49] Vlachopoulos S.P., Katartzi E.S., Kontou M.G., Moustaka F.C., Goudas M. (2011). The revised perceived locus of causality in physical education scale: Psychometric evaluation among youth. Psychol. Sport Exerc..

[50] Vallerand R.J. (2007). Intrinsic and extrinsic motivation in sport and physical activity. Handb. Sport Psychol..

[51] Hein V., Müür M., Koka A. (2004). Intention to be physically active after school graduation and its relationship to three types of intrinsic motivation. Eur.Phys. Educ. Rev..

[52] Moreno J.A., Moreno R., Cervelló E. (2007). El autoconcepto físico como predictor de la intención de ser físicamente activo. Psicología y Salud..

[53] Hair J.F., Black W.C., Babin B.J., Anderson R.E., Tatham R.L. (2006). Multivariate Data Analysis.

[54] Marsh H.W., Hau K.T., Wen Z. (2004). In search golden rules: Comment on hypothesis-testing approaches to setting cutoff values for fit indexes and dangers in overgeneralizing Hu and Bentler’s (1999) findings. Struct. Equ. Modeling.

[55] McDonald R.P., Ho M.H.R. (2002). Principles and practice in reporting structural equation analyses. Psychol. Methods..

[56] Salami S.O. (2010). Emotional intelligence, self-efficacy, psychological well-being and students attitudes: Implications for quality education. J. Stud. Int. Educ..

[57] Cañabate D., Martínez G., Rodríguez D., Colomer J. (2018). Analysing emotions and social skills in physical education. Sustainability..

[58] Magnano P., Craparo G., Paolillo A. (2016). Resilience and Emotional Intelligence: Which role in achievement motivation. Int. J. Psychol. Res..

[59] Kusurkar R.A., Ten-Cate T.J., Vos C.M.P., Westers P., Croiset G. (2013). How motivation affects academic performance: A structural equation modelling analysis. Adv. Health Sci. Educ..

[60] Sun H., Chen A. (2010). An examination of sixth graders’ self-determined motivation and learning in physical education. J. Teach. Phys. Educ..

[61] Vansteenkiste M., Simons J., Lens W., Sheldon K.M., Deci E.L. (2004). Motivating learning, performance, and persistence: The synergistic effects of intrinsic goal contents and autonomy-supportive contexts. J. Pers. Soc. Psychol..

[62] Trigueros R., Navarro N. (2019). La influencia del docente sobre la motivación, las estrategias de aprendizaje, pensamiento crítico de los estudiantes y rendimiento académico en el área de Educación Física. Psychol. Soc. Educ..

[63] Berkowitz R., Moore H., Astor R.A., Benbenishty R. (2017). A research synthesis of the associations between socioeconomic background, inequality, school climate and academic achievement. Rev. Educ. Res..

[64] Zeng G., Hou H., Peng K. (2016). Effect of growth mindset on school engagement and psychological well-being of Chinese primary and middle school students: The mediating role of resilience. Front. Psychol..

